# Impact of the COVID-19 Pandemic on Patients With Alcohol Use Disorder and Associated Risk Factors for Relapse

**DOI:** 10.3389/fpsyt.2020.620612

**Published:** 2020-12-16

**Authors:** Kurosch Yazdi, Isabella Fuchs-Leitner, Jan Rosenleitner, Nikolas W. Gerstgrasser

**Affiliations:** ^1^Department of Psychiatry - Specialization Addiction Medicine, Kepler University Hospital, Linz, Austria; ^2^Faculty of Medicine, Johannes Kepler University Linz, Linz, Austria

**Keywords:** COVID-19, alcohol use disorder (AUD), relapse, psychosocial impact, PTSD symptom, isolation

## Abstract

**Background:** The impact of the ongoing COVID-19 pandemic on vulnerable groups like patients suffering from substance use disorders is expected to be tremendous, and corresponding concerns were raised early on by many experts around the world. Psychosocial distress, financial insecurities and physiological problems associated with the COVID-19 crisis could be especially challenging for this group of patients.

**Methods:** In the current study data was collected from a clinical sample of patients with alcohol use disorder (AUD; *N* = 127) during the initial stage of the pandemic. The impact of various COVID-19 related factors (physiological, psychosocial, economic and others) on patients' personal life was evaluated. Alcohol consumption, craving, and potential posttraumatic stress disorder (PTSD) symptoms were assessed using different scales and their associations were analyzed. Furthermore, differences regarding these variables between comparably sized groups of patients who remained abstinent (*N* = 37), relapsed (*N* = 41), or reported unaltered drinking behavior (consuming subgroup, *N* = 49) were investigated. The impact of sociodemographic and COVID-19 factors on relapse (in comparison to abstinence) was evaluated using binary logistic regression analysis.

**Results:** Our results confirmed the expected positive associations between alcohol consumption, craving, and PTSD symptoms, respectively, among patients with AUD. Furthermore, group differences indicate significantly lower levels on all three scales for abstinent patients. Although generally low PTSD scores were observed, 8% of our participants were found to be at risk of PTSD. Results of a binary logistic regression analysis indicated the presence of psychosocial COVID-19 factors (e.g., isolation, anxiety, and depression) as well as living alone as two major risk factors for relapse.

**Discussion:** Our findings based on actual patient data support the anticipated negative consequences of the pandemic on persons with AUD. Crucially, our results regarding relapse emphasized psychosocial COVID-19 factors and isolation as especially challenging circumstances for persons with AUD, whereas economic and physiological health aspects seemed of minor impact on relapse. Our results reflect the initial stage of the pandemic, whereas long-term developments should be closely monitored.

## Introduction

The current pandemic with a novel corona virus, SARS-CoV-2 (severe acute respiratory syndrome coronavirus 2), and its worldwide spreading is extensively impacting on the global physical and mental health. At the end of 2019, a cluster of atypical cases of pneumonia was observed in Wuhan, Hubei Province, China (1), which shall be designated as *Coronavirus disease 2019* (COVID-19) by the World Health Organization (WHO) on February 11, 2020 (2). Rapidly evolving, on March 11, 2020, the WHO made the assessment that the outbreak could be characterized as a global pandemic (2). The reported symptoms of COVID-19 are primarily respiratory with acute respiratory distress syndrome ultimately leading to death in the most severe cases (3). Effects on other organs, including the brain, and neurological symptoms due to COVID-19 infection have been recently reported [for a recent review see Vindegaard et al. (4)].

Alongside the obvious physiological impact of COVID-19, economic, psychosocial and other COVID-19 related factors immensely affect further areas of life during this ongoing pandemic. From an economic perspective, social distancing, self-isolation and travel restrictions have led to a reduced workforce across all economic sectors (5). Hence, insolvent businesses, job losses and financial insecurities are unavoidable consequences. Taking that into account, the economic impact of the COVID-19 pandemic seems to be a substantial source of distress.

Psychosocial impact of the pandemic is far-ranging, and increases in stress, anxiety, depressive symptoms, sleep disorders, denial, anger, and fear, have been clearly articulated (6, 7). Short-term and long lasting mental health impacts of COVID-19 on the general population are not yet quantifiable, but are expected to be tremendous (8). COVID-19 associated government measures like physical distancing and the uncertainty about future development additionally worsen the prospects of mental health issues (9). The psychological impact of quarantine was reviewed in detail by Brooks et al. (10).

In particular, the current pandemic and its related psychological stressors are expected to promote PTSD due to COVID-19 as a common psychiatric response (11). A high prevalence of posttraumatic stress was evident in China's hardest-hit areas 1 month after the COVID-19 outbreak (12). The COVID-19 pandemic is associated with significant levels of psychological distress in the general population (13, 14). Similar findings were reported in Italy (15).

Although the COVID-19 crisis is unique in many aspects, studies on former pandemics (e.g., SARS outbreak in China in 2003) implicate higher levels of stress and psychological distress among SARS survivors during and even 1 year after the outbreak (16). In this context, distress was a frequently observed symptom in the general Chinese population with up to 35% during the initial phase of the COVID-19 pandemic (17). Data from an anonymous online questionnaire survey showed a prevalence of PTSS of 4.6% in mainland China 1 month after the outbreak of the virus (18). 14.6% of participants of an Italian survey (15) were in the high range and 12.6% in the extremely high range according to the stress subscale of the *Depression, Anxiety and Stress Scale*−*21 items* (DASS-21) (19). In a recent study, Di Crosta et al. found that 35.6% (*N* = 446) scored above the cutoff score on the *Impact of Event-Scale – Revised* (IES-R) (20) and thus belonged to the high-PTSD group (21). This high number of participants at risk of PTSD in the general population is alarming. Limited access to mental health services during the pandemic may even deteriorate the situation, and global strategies are indispensable facing the related mental health issues.

### Impact of COVID-19 on Addictive Behaviors and Disorders

Unsurprisingly, the COVID-19 pandemic can alter pre-existing or trigger new addictive behaviors. In this context, an increased prevalence (4.3%) of severe Internet use disorder, as well as rising numbers of relapse in alcohol (19%) and smoking abuse (25%) were reported (22). These three behaviors were interpreted as coping strategies during this crisis. As anticipated, distress (especially during long periods of isolation) resulting from this pandemic may result in negative emotions and related maladaptive coping styles (23). However, results from various European studies on the general population indicated both, increases and decreases in alcohol consumption. According to an UK-survey 21% of the participants reported to drink alcohol more frequently and 15% to drink more alcohol per session during the lockdown than before. In the subgroup of daily drinkers 18% increased their amount of alcohol (24). The same study reported that a third stopped drinking or reduced their frequency since the lockdown in March, whereas 6% ceased drinking alcohol entirely (24). A study from Poland even found that alcohol was the most commonly used psychoactive substance in this country (almost 73%), followed by tobacco smoking (25%) during the initial stage of the pandemic (25). According to this survey, 14% of the participants reported to drink more alcohol, whereas 16% consumed less alcohol than pre-epidemic. An Austrian study reported an increase in alcohol consumption in 14% of participants and 2% even just starting to drink alcohol due to the COVID-19 crisis (26).

The COVID-19 crisis might affect vulnerable persons particularly hard (27). Physiological aspects in this context might be even more distressing among this group, since marginalized communities—especially those with substance use disorder (SUD) (28)—are at greater risk of worse COVID-19 outcome (29). Pre-existing cardio-pulmonary morbidities, compromised immunity, mucociliary dysfunction and altered health-seeking behavior might additionally increase the risk of infection for patients with SUDs [for an overview see Dubey et al. (29)]. An overall worse health condition and damaging effects of drugs on the cardiovascular system might further increase the risk of mortality associated with COVID-19 (28, 30). Anticipated psychological consequences of the pandemic, including depression, anxiety, irritability and anger among persons suffering from SUD, are expected to heighten the risk for relapse into a new episode of drug use (28).

In respect to alcohol use disorder (AUD), alcohol consumption leads to a significantly higher risk for contracting bacterial and viral lung infections (including COVID-19) (31). Psychosocial distress might be particularly challenging for patients with SUDs, since social distancing and quarantine might intensify isolation and loneliness (32). In this context, living alone is associated with a greater risk of suffering from SUDs in older adults (33). Furthermore, family support was emphasized to play a crucial role in preventing relapse of persons with addiction problems (23), challenging especially for those patients who were living alone during lockdown phases. Economic aspects including job loss might worsen potential preexisting financial troubles and poverty (30). In fact, studies on economic crises found associations between an increase in unemployment with a substantial increase (28%) in mortality due to SUDs and higher numbers of suicide (4.5%) (34). Additionally, the pandemic disproportionately affects people with SUDs by diminishing resources that people with SUD need for their recovery and wellbeing (32).

Combining these aspects, deterioration of preexisting conditions such as AUD and associated relapse were anticipated (30). In general, pre-existing mental disorders (including SUDs) increase the risk of relapse during the pandemic (27). A recent study from China reported almost a fifth (18.7%) of abstinent persons suffering from AUD who relapsed during the first phase of the pandemic, and about a third of regular drinkers increased the amount of consumed alcohol (22). In line with these findings, a study from the UK observed that 17% of former abstinent patients relapsed during lockdown (35). Naturally, addiction psychiatry is facing major challenges during this pandemic to maintain high standards in care (36).

A recent study on the impact of the COVID-19 pandemic on various addictive disorders in Italy found relatively high rates of depression, anxiety, irritability, and posttraumatic stress symptoms among a clinical sample of patients suffering from different SUDs (including alcohol, cocaine and THC). Furthermore, the authors evaluated quality of life and craving in this context (37). Craving is one of the key symptoms and predictor for relapse in patients with addictive disorders (38). They found positive associations between craving with symptoms of depression, anxiety, and traumatic stress (37). Associations between stress and anxiety levels with increased alcohol use during the initial stage of the pandemic have already been demonstrated (39). Furthermore, addictive disorders and PTSD seem to be interconnected (40), and AUD and PTSD are both known outcomes of former crises (41).

### COVID-19 Situation in Austria

**Figure 1:**
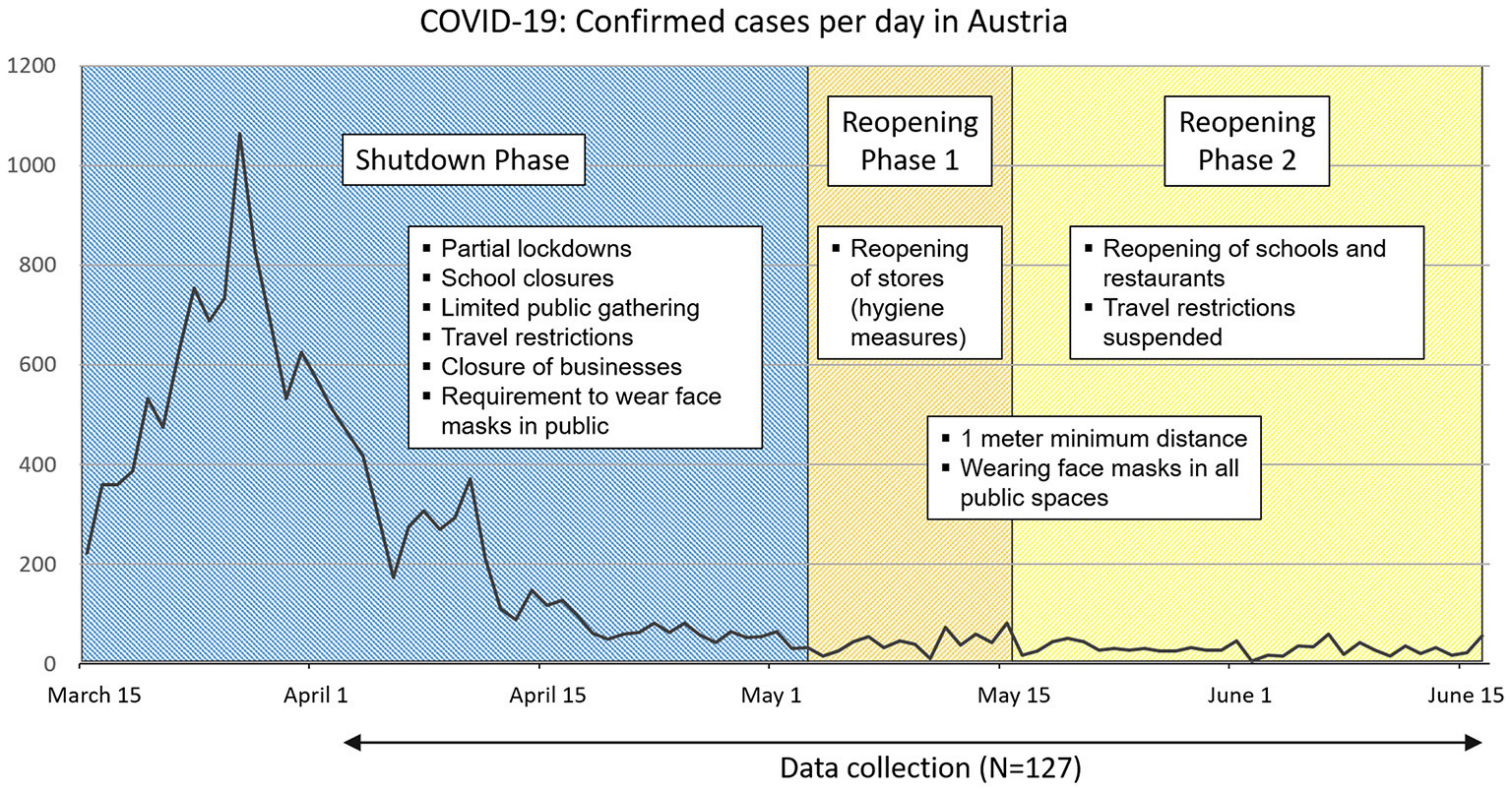
Incidence of COVID-19 cases in Austria and data collection of the study. The confirmed cases of COVID-19 in Austria^1^ (total population of 8.859 Million) between mid-March and mid-June 2020 are displayed. Examples of related government measures during the shutdown and reopening phases and the period of the data collection are provided.

Incidents of confirmed COVID-19 cases in Austria (total population of 8.859 Million) between March and June 2020 are displayed in Figure 1. The first case of COVID-19 was confirmed on February 25, 2020. The government responded to the quick increase of cases in mid-March with massive restrictions and a shutdown phase including partial lockdowns. After a drop in COVID-19 cases, the first reopening phase began at the beginning of May with the reopening of stores and services under strict hygiene measures. The next reopening phase mid-May included the reopening of schools and restaurants, as well as the suspension of travel restrictions and border openings. The development of COVID-19 cases and mortality during this initial phase was comparable to other European countries like Germany or Switzerland.

### Aims and Research Questions

Concerns about the multifaceted consequences of the pandemic on patients with SUDs were raised early in this pandemic (29, 32). However, studies including clinical populations are rare so far. The current study therefore aimed to investigate addictive behavior, craving, and PTSD symptoms, as well as various COVID-19 factors directly in a clinical sample of patients with AUD during the initial stage of the pandemic.

First, associations between current alcohol consumption (i.e., frequency, quantity and heavy drinking days), subjective craving and PTSD-symptoms were assessed. Second, differences regarding these aspects between groups of patients who remained abstinent, relapsed, or showed unaltered alcohol consumption behavior (i.e., were still consuming) after the onset of COVID-19 in Austria were evaluated. Third, the impact of different sociodemographic and COVID-19 related factors on relapse (vs. abstinence) during the beginning of the pandemic were investigated.

## Materials and Methods

### Participants

Data was collected from patients diagnosed with AUD (*N* = 127) at our inpatient and outpatient facilities as part of routine anamneses. This study includes a retrospective data analysis and was conducted in accordance to the Declaration of Helsinki and approved by the local ethics committee. Data was processed and analyzed anonymously. All patients seeking help at our facilities between the beginning of April and mid-June 2020, who were diagnosed with AUD and consented to provide their responses were included in this study. From our total sample 41.7% were treated at our inpatient facilities. Outpatients were assessed either in face-to-face consultations (24.4%) or via telephone (33.9%).

According to their current state of alcohol consumption with respect to the beginning of the COVID-19 pandemic in Austria, participants were classified into three subgroups: persons remaining abstinent (*N* = 37), patients suffering relapse since mid-March (*N* = 41) and those still consuming (unaltered since COVID-19; *N* = 49). Descriptive summary statistics of sociodemographic variables of the total sample and the three subgroups are shown in Table 1.

**Table 1:** Sociodemographic factors of the total sample and the three subgroups respectively.

	**Total (*N* = 127)**	**Abstinent (*N* = 37)**	**Consuming (*N* = 49)**	**Relapsed (*N* = 41)**
	**Percent/Mean (SD)**	**Percent/Mean (SD)**	**Percent/Mean (SD)**	**Percent/Mean (SD)**
**Sociodemographic factors**				
Age (in years)	49.3 (12.3)	51.0 (13.0)	48.5 (13.4)	48.9 (10.5)
Gender: Male	66.9%	64.9%	63.3%	73.2%
Living alone	42.5%	29.7%	40.8%	56.1%
Outdoor space available	83.5%	86.5%	85.7%	78.0%

*SD, standard deviation*.

*For interval data mean and standard deviation are presented; for dichotomous variables the percentage of the given subset indicating “yes” is displayed*.

### Procedure

Data was collected shortly after the onset of the COVID-19 crisis in Austria for 10 weeks (between the beginning of April until mid-June 2020, see also Figure 1). Relevant sociodemographic information (e.g., age, gender, living alone, access to outdoor spaces during the lockdown) was collected as part of routine anamneses.

Current alcohol consumption was assessed by the German version of the *Alcohol Use Disorder Identification Test* (consumption part: AUDIT-C) (42). The AUDIT (43) is a widely used screening tool developed by the World Health Organization (WHO). The short version AUDIT-C consists of the first three questions of the AUDIT and relates to alcohol consumption (frequency, quantity, and heavy drinking days) with a total range from 0 to 12. To identify alcohol misuse, screening thresholds of 4 (in men) and 3 (in women) are recommended. Subjective craving was indicated by the patients on a 5-point Likert scale (ranging from 0 = no craving at all to 4 = intense craving).

To evaluate the presence of PTSD and indicated stress symptoms triggered by COVID-19 the German version of the *Primary Care PTSD screen for DSM5* (PC-PTSD5, range 0–5) (44) was used. The screening tool consists of five questions about how a traumatic event has affected the patient over the past month. These questions correspond to DSM-5 criteria for PTSD and include typical symptoms like re-experiencing, numbing, avoidance, hyperarousal, and guilt. Patients were asked to respond exclusively with respect to the COVID-19 pandemic and related government measures as a potential traumatic event.

The impact and burden of COVID-19 related factors on patients' personal life was evaluated. To that end, patients were asked to determine the presence or absence of different aspects in regard to the COVID-19 pandemic that resulted in personal worries or problems. The corresponding answers were categorized into four different COVID-19 factors. Physiological aspects included all health problems, as well as access to health care in relationship to the COVID-19 pandemic. Economic factors ranged from financial problems, economic uncertainty to job loss due to the pandemic. Psychosocial aspects included negative emotions such as depression, fear, anxiety, and worries about others, as well as a reported psychological burden as a result of isolation during this initial stage and lockdown. A reported lack of access to alcohol, as well as closing of bars were summarized as other factors. Each of these four COVID-19 factors was registered as either absent or present for each participant.

### Statistical Analysis

Data was analyzed using IBM SPSS Statistics for Windows (Version 25.0) (45). Descriptive statistics such as (relative) frequencies for nominal variables are presented. Ordinal and metric variables are described using the median and the interquartile range or the mean and the standard deviation, respectively. To assess potential associations between the scale scores (i.e., alcohol consumption, craving, and PTSD), spearman rank correlations were calculated for the total sample. Group differences in the scale scores across abstinent, relapsed, and consuming patients were investigated using Kruskal-Wallis tests. Sociodemographic variables as well as COVID-19 factors were analyzed as possible risk factors for relapse, using a binary logistic regression model for the outcome variable relapse (comparison of relapsed and abstinent patients). The significance level is defined as 0.05. Hence, small *p*-values indicate possible associations between the variables. Detailed information on the applied analyses can be found in the Results section below.

## Results

### Descriptive Statistics

Descriptive statistics for COVID-19 factors, as well as alcohol consumption (AUDIT-C), craving, and PTSD symptoms (PC-PTSD5) are displayed for the total sample and the three subgroups (abstinent, relapsed and consuming patients) in Table 2.

**Table 2:** Descriptive statistics for COVID-19 factors and scales in the total sample and the three subgroups respectively.

	**Total (*N* = 127)**	**Abstinent (*N* = 37)**	**Consuming (*N* = 49)**	**Relapsed (*N* = 41)**
	**Percent**	**Percent**	**Percent**	**Percent**
**COVID-19 factors**				
Physiological factors	24.4%	27.0%	22.5%	24.4%
Economic factors	21.3%	21.6%	16.3%	26.8%
Psychosocial factors	53.5%	32.4%	59.2%	65.9%
Other factors	21.3%	18.9%	24.5%	19.5%
	**Median (IQR)**	**Median (IQR)**	**Median (IQR)**	**Median (IQR)**
**Scales**				
AUDIT-C (0–12)	7 (12)	0 (0)	10 (6)	11 (6)
Craving (0–4)	2 (2)	1 (2)	2 (2)	3 (3)
PC-PTSD5 (0–5)	0 (1)	0 (0)	0 (1)	0 (2)

*IQR, interquartile range; For dichotomous variables the percentage of the given subset indicating “yes” is displayed; for ordinal data median and interquartile range are given*.

In the total sample (mean age = 49.3 years, 66.9% male), psychosocial COVID-19 factors were reported by the majority of patients (53.5%), whereas burden by physiological, economic and other factors were indicated less frequently (between 21.3 and 24.4%).

Alcohol consumption measured by AUDIT-C scores (range 0–12) were high in the relapsed (median = 11) and consuming (median = 10) subgroups of patients. Craving scores (range 0–4) were also highest among those who relapsed (median = 3) at the initial stage of the pandemic. Regarding PTSD symptoms due to COVID-19, only a third of our patients (31.7%) reported one or more symptoms, resulting in medians of zero for the total sample and the three subgroups. Importantly, 7.9% (*N* = 10) of the sample were indicated at risk of PTSD due to the pandemic (with a recommended PC-PTSD5 cut-off score of 3 or more) (44). Half of these patients were in the relapsed group, four were in the consuming group and only one patient was in the abstinent group.

### Association Between Alcohol Consumption (AUDIT-C), Craving, and PTSD Symptoms (PC-PTSD5)

**Figure 2:**
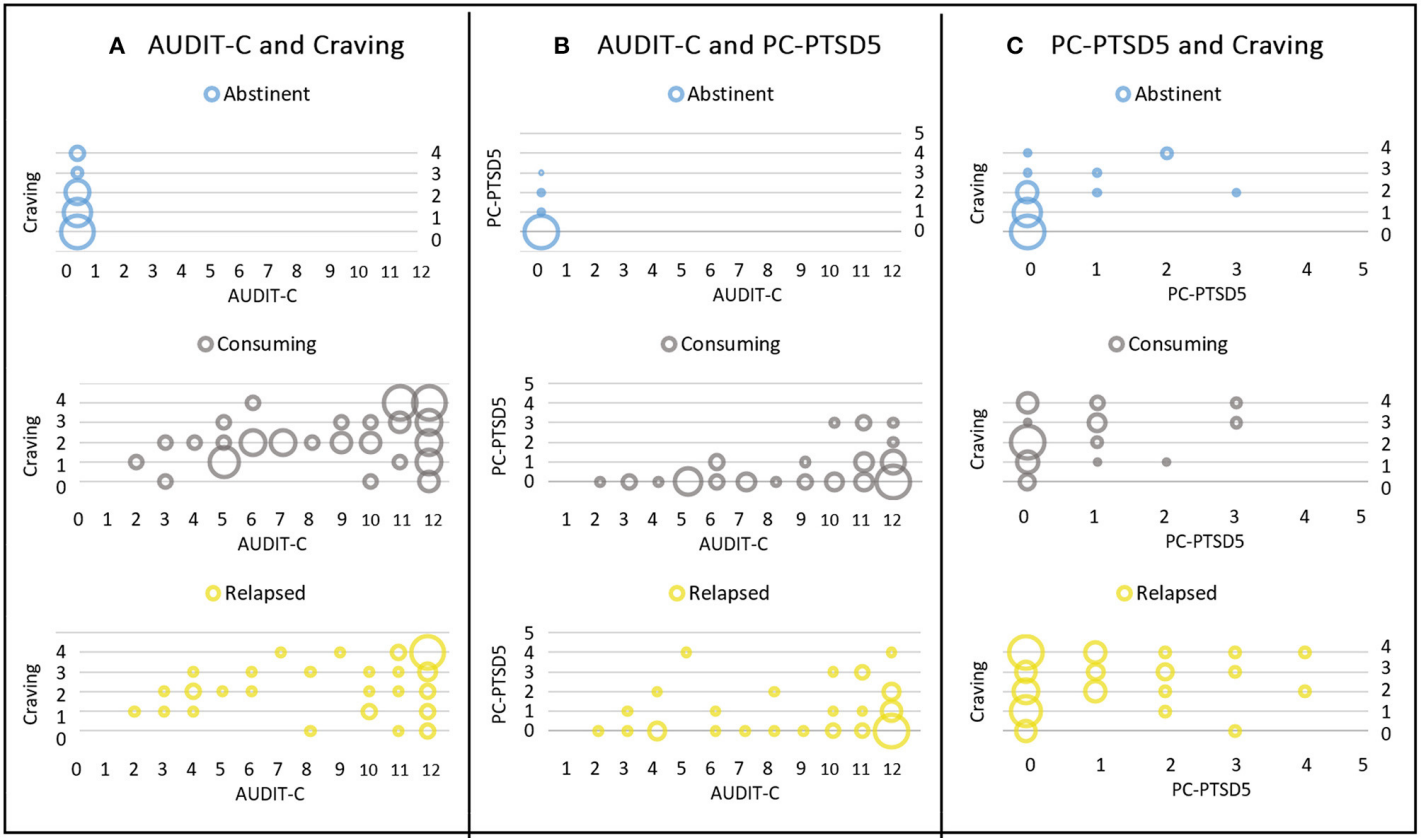
Bubble plots of different combinations of the three scales. Results are presented for the three subgroups of abstinent (first row), consuming (second row), and relapsed patients (third row). **(A)** Depicts AUDIT-C (x-axis) and craving scores (y-axis), **(B)** AUDIT-C (x-axis) and PC-PTSD5 scores (y-axis), and **(C)** shows PC-PTSD5 (x-axis) and craving scores (y-axis). Scores of different subgroups are depicted in blue (abstinent), gray (consuming), and yellow (relapsed group). The size of the bubble represents the number of cases, i.e., bigger bubbles indicate higher numbers of participants with the respective combination of scores.

Spearman rank correlations between alcohol consumption (AUDIT-C) and craving and PTSD symptoms (PC-PTSD5) were calculated for the total sample. (Please note that all abstinent patients scored zero on the AUDIT-C.) Significant positive correlations between all three factors were found, with moderate correlations for AUDIT-C and craving (Spearman's rho = 0.44, *p* < 0.001) and AUDIT-C and PC-PTSD5 scores (Spearman's rho = 0.41, *p* < 0.001), respectively. Craving and PC-PTSD5 scores showed a weak to moderate positive correlation (Spearman's rho = 0.29, *p* = 0.001). These results suggest a positive association between all three variables, indicating higher levels of alcohol consumption (AUDIT-C score) with higher levels of stress (PC-PTSD scores) and craving. Bubble plots of the different combinations of scales, and for the three subgroups are depicted in Figure 2.

### Groupwise Comparisons for Alcohol Consumption, Craving and Stress

**Figure 3:**
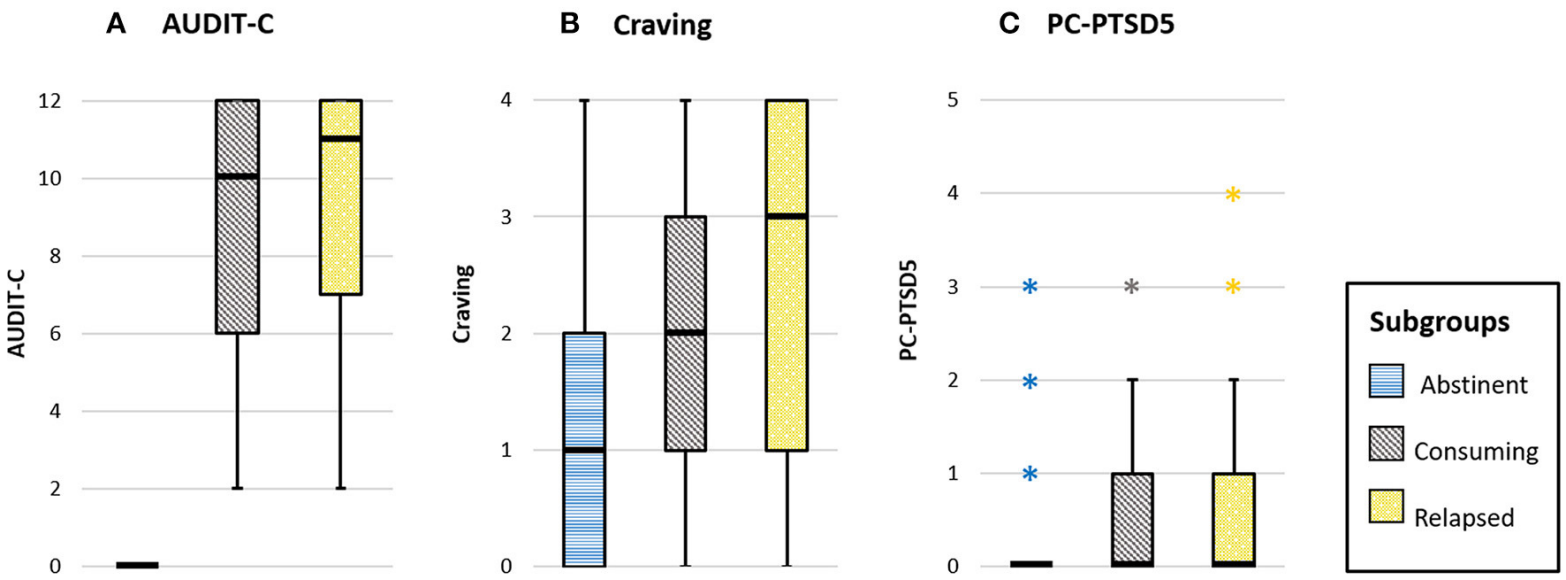
Boxplots for the scales **(A)** AUDIT-C (range 0–12), **(B)** craving (range 0–4), and **(C)** PC-PTSD5 (range 0–5) are provided for the three subgroups. The different subgroups are depicted in blue (abstinent), gray (consuming), and yellow (relapsed group). Outliers are presented as asterisks.

AUDIT-C, craving, and PTSD scores were compared between the three groups of abstinent, relapsed, and consuming patients, respectively, using Kruskal-Wallis tests. Boxplots for the scales per subgroup are shown in Figure 3.

A significant difference between the three groups was found for AUDIT-C scores, *H* (2) = 82.1, *p* < 0.001, *d*_*COHEN*_ = 2.7. *Post-hoc* Dunn-Bonferroni groupwise tests showed significant differences between the abstinent group and both relapsed (*p* < 0.001) as well as consuming patients (*p* < 0.001), respectively. Note that all patients in the abstinent group scored zero on the AUDIT-C. This finding therefore indicates the obviously higher alcohol consumption scores for the two other subgroups. Relapsed and consuming patients did not differ with respect to AUDIT-C scores.

For craving, a significant difference between the groups was found, *H*(2) = 19.4, *p* < 0.001, *d*_*COHEN*_ = 0.81. *Post-hoc* Dunn-Bonferroni groupwise comparisons revealed significant differences for the abstinent group compared to both relapsed (*p* < 0.001) and consuming patients (*p* = 0.001; consuming vs. relapsed: *p* = 1.0), respectively. These findings indicate lower subjective craving for the abstinent compared to the other patients (see also descriptive statistics in Table 2).

A significant difference between the three groups was also found for the PTSD scores, *H*(2) = 8.6, *p* = 0.013, *d*_*COHEN*_ = 0.47. *Post-hoc* performed Dunn-Bonferroni tests revealed a significant difference only between abstinent and relapsed patients (*p* = 0.01), but not between the other groups (abstinent vs. consuming: *p* = 0.26; relapsed vs. consuming: *p* = 0.50). These results suggest higher subjective stress (corresponding to higher PTSD scores) for relapsed patients compared to the abstinent group (see also descriptive statistics in Table 2).

### Modeling and Predicting Relapse With Logistic Regression Analysis

Binary logistic regression analysis was performed for the subsample (*N* = 78) of patients being abstinent before the beginning of the pandemic, and either remained abstinent (*N* = 37) or relapsed (*N* = 41) throughout the initial stage of COVID-19. The model allows to evaluate the effects of sociodemographic factors (age, gender, living alone, access to outdoor spaces) and COVID-19 impact (physiological, economic, psychosocial and other factors) on the probability of relapse. A backward variable selection procedure (Wald) was performed using a cutoff value of 0.53 (i.e., the proportion of relapsed patients in this subsample). Results of this regression analysis are presented in Table 3 in form of the full model and the final model after variable selection.

**Table 3:** Results of the binary logistic regression model for relapsed (vs. abstinent) patients.

	**B**	**SE**	**Wald χ^2^**	**OR**	**95% CI**	***p***
**Initial Model (Step 1)**						
Age	−0.03	0.02	1.56	0.97	0.93–1.02	0.212
Gender	−0.28	0.57	0.24	0.76	0.25–2.32	0.627
Living alone	1.05	0.58	3.30	2.86	0.92–8.89	0.069
Outdoor space	0.04	0.74	0.00	1.04	0.25–4.43	0.953
Physiological Factors	−0.30	0.60	0.26	0.74	0.23–2.37	0.609
Economic Factors	0.06	0.62	0.01	1.07	0.32–3.59	0.918
**Psychosocial Factors**	1.42	0.53	7.20	4.13	1.47–11.61	**0.007**
Other Factors	−0.37	0.69	0.28	0.70	0.18–2.68	0.595
Constant	0.62	1.42	0.19	1.86		0.663
	**B**	**SE**	**Wald χ^2^**	**OR**	**95% CI**	***p***
**Final Model (Step 6)**						
Age	−0.03	0.02	1.87	0.97	0.93–1.01	0.171
Gender	^*^	^*^	^*^	^*^	^*^	^*^
**Living alone**	1.10	0.53	4.36	3.00	1.07–8.39	**0.037**
Outdoor space	^*^	^*^	^*^	^*^	^*^	^*^
Physiological Factors	^*^	^*^	^*^	^*^	^*^	^*^
Economic Factors	^*^	^*^	^*^	^*^	^*^	^*^
**Psychosocial Factors**	1.30	0.50	6.63	3.65	1.36–9.79	**0.010**
Other Factors	^*^	^*^	^*^	^*^	^*^	^*^
Constant	0.53	1.11	0.23	1.69		0.634

*Results and test statistics for the initial and final logistic regression model (step 6) are displayed. Significant results with p < 0.05 are presented in bold letters*.

*SE, standard error; OR, odds ratio; CI, confidence interval*.

* *Variables dropped in backward selection procedure*.

The model with the highest correct classification rate (step 6 of 7: 70.5%) was selected as the final logistic regression model. ^2^ This final model included psychosocial COVID-19 factors, age, and living alone as predictors, and was statistically significant, χ^2^(3) = 14.3, *p* = 0.003. Nagelkerke *R*^2^ of 22.4% shows a moderate goodness of fit of the model, which has high levels of sensitivity (0.78) and specificity (0.62). Patients with psychosocial COVID-19 factors have an increased risk (odds ratio=3.65, *p* = 0.010) of relapsing compared to patients not reporting psychosocial impact of COVID-19. Living alone also leads to a higher risk of relapsing (odds ratio of 3.00, *p* = 0.037) compared to those living with others, and age showed a small negative non-significant effect (odds ratio = 0.97, *p* = 0.171).

## Discussion

The current study investigated different aspects of COVID-19 in a clinical sample of persons with AUD, who sought help at our inpatient and outpatient facilities during the initial stage of the pandemic. Furthermore, although the impact of the COVID-19 crisis might differ between individuals, we aimed to identify general risk factors regarding relapse of persons with AUD. Current alcohol consumption, subjectively perceived craving, and PTSD symptoms were assessed as relevant factors for AUD with respect to COVID-19. A general increase regarding addictive behavior due to COVID-19 was anticipated and already confirmed for a Chinese population (18). Specifically, increased alcohol consumption was reported during the initial stage of the pandemic in different European countries (24, 25), including Austria (26). However, corresponding data from persons with AUD is still lacking. In our clinical sample, alcohol consumption was reported to be rather high among consuming and relapsed patients (with median scores of 10 and 11 compared to a maximum of 12 on the AUDIT-C, respectively). Regarding craving, a moderate level was found in the total sample. PTSD scores were generally low, with two thirds of our patients not reporting any PTSD symptoms due to COVID-19 at all.

In line with our first aim, anticipated associations between the three variables alcohol consumption, craving and posttraumatic stress symptoms were confirmed. It is not surprising that increased craving—irrespective of its cause—leads to increased alcohol consumption (46). On the other hand, alcohol consumption can lead to increased craving via feedback loops of the reward system as described by the term addiction cycle (47). The association between alcohol consumption and PTSD symptoms is in line with prior findings reporting the interconnection between PTSD and SUDs (40). Furthermore, the positive correlation between craving and PTSD symptoms was also reported in a recent study on persons with SUDs (37). The authors also stress the importance to consider associations between craving and psychopathological conditions to gain useful information for successful treatment and prevention strategies.

Our clinical sample consisted of three subgroups of patients who remained abstinent, relapsed, or were consuming before and after the onset of the pandemic. The second aim of this study was to further investigate group differences regarding the various scores. Naturally, lower alcohol consumption (i.e., a score of zero) was reported among abstinent persons compared to the other subgroups. Craving was also significantly lower for abstinent compared to both, relapsed and consuming patients. One can only speculate about the causal relationships. However, an increase in craving scores has already been described by other authors to be associated with an elevated risk for relapse (38). We found significant differences between abstinent and relapsed patients for PTSD scores. Though PTSD did not affect most patients in our sample, we also found 8% of the sample at risk of PTSD due to the pandemic, whereof the majority was part of the relapsed subgroup. This finding indicates that those at risk of PTSD seemed to be at risk of drinking, too. Screening via PC-PTSD-5 at any contact with AUD would thus be helpful during the ongoing crisis, since this questionnaire is short and can easily be implemented into any routine anamnesis. The COVID-19 pandemic does cause traumatic stress for a substantial portion of people suffering from SUD and these persons need special attention by providers of addiction treatment. Otherwise, they are at high risk of relapse or to continue drinking with standard SUD care without focus on PTSD falling short.

Our final aim was to investigate different sociodemographic and COVID-19 factors as potential risk factors for relapse among persons with AUD. Recent literature discussed the potential harming effects of various relevant aspects of life due to COVID-19. Physiological factors involve the elevated risk of a severe outcome of COVID-19 among persons with AUD (31). Most prominently, psychosocial factors like depression, anxiety and isolation are discussed to impact not only the mental health of the general population (6, 7), but are expected to be especially severe for persons with SUDs (32). Economic aspects during the COVID-19 pandemic are anticipated to be particularly challenging for persons with addictive disorders (30). A binary logistic regression model revealed significant impacts of psychosocial COVID-19 factors and living alone, and a small non-significant negative effect of age as increasing the probability for relapse in AUD. Distressing psychosocial factors even manifested as psychiatric comorbidities (e.g., depression) are generally common in SUDs, but the COVID-19 situation has intensified these burdening factors. As they seem to be of predictive value, they need to be considered especially for abstinent patients to make relapses less likely during the ongoing pandemic. Our finding that living alone increased the probability for relapse is also in line with literature emphasizing the importance of family support in preventing relapse (23). Furthermore, living alone was found to be associated with a generally higher risk for SUDs in a sample of persons aged 50 years and older (33).

Based on our current findings, abstinent persons suffering from AUD, who are living alone and report the presence of psychosocial distress due to COVID-19 should be in special focus of health care providers with respect to potential relapse. Complementary measures to support this group through the pandemic could be telemedicine services for diagnostic purposes as well as counseling (48). Our results further indicate that physiological and economic aspects of COVID-19 do not seem to play a crucial role as risk factors for relapse in AUD, at least during the study period. This is surprising, given the fact that many persons in our sample have considerable somatic comorbidities and are heavy users of different health services under usual circumstances, where parts of those services were not easily accessible during the experienced lock down. Furthermore, our data does not support anticipated concerns of other authors regarding particular distress stemming from economic and financial problems (30). One reason for this discrepancy could be due to the early stage of the COVID-19 crisis at the time of our data collection between April and June 2020. Back then, most Austrians expected the pandemic to be over soon, and the government provided substantial financial support for companies to prevent massive job losses. Thus, people might have been optimistic about the outcome of the crisis and their personal situation at that time. With the progression of the pandemic the worries about the individual economic and health situation could have changed though. On the long term, this might be a cause of considerable distress and might even promote relapses in AUD.

Our findings involve some limitations, and have to be interpreted with caution. First, the current study investigated individual-level characteristics, whereas area-level correlates (e.g., levels of education, unemployment, or overcrowding in a specific geographical area) (49) were not evaluated. Since our findings are deflected from patients living in the same region (i.e., Upper Austria) and more detailed information (e.g., district of residence) was not assessed, potential impact of unexplored area-level factors cannot be excluded. Established associations between area-level deprivation and adverse consequences of SUDs (49) might also play a crucial role for relapse in AUD. Hence, these variables should be taken into account in future studies. Second, as the data in the current study was collected at a specific point in time (i.e., during the first stage of the COVID-19 pandemic) it has to be considered a cross-sectional study. Naturally, limitations of this type of study also apply for the current findings. Since exposure and outcome are assessed at the same time, interpretations of the temporal relationships between cause and effect without longitudinal data are restricted. Consequently, the direct impact of the identified risk factors for relapse in AUD have to be evaluated. Further investigations are therefore inevitable to fully understand the long-term consequences of the pandemic. Third, the clinical sample investigated in this study qualifies as “convenience sampling,” and leads to another limitation. Since our findings are solely based on patients with AUD, conclusions about the general population cannot directly be drawn.

In conclusion, our data suggests that the current situation and specially periods of COVID-19 caused lockdowns overstrain the capacity of stress management and relapse prevention as a substantial part of this vulnerable group suffering from AUD. Without quick and specific help by health care services many of them would use alcohol as means of short-termed stress management. Conceiving psychosocial stressors and PTSD symptoms should be part of every inpatient or outpatient contact and depending on their incidence the medical care should be intensified. But also the health care system as a whole should lay particular attention on SUD, since this group needs extra support due to the crisis on hand. In case of further lockdowns people suffering from SUD need unhindered and low-threshold access to treatment. However, our data only depicts the first phase of the COVID-19 pandemic including the first lockdown stage. More research is needed to capture long-term effects and to develop long-acting strategies for the support of persons with SUDs during this ongoing and future pandemic.

## Data Availability Statement

The raw data supporting the conclusions of this article will be made available by the authors, without undue reservation.

## Ethics Statement

The studies involving human participants were reviewed and approved by Ethics Commission of the Medical Faculty of the Johannes Kepler University Linz. Written informed consent for participation was not required for this study in accordance with the national legislation and the institutional requirements.

## Author Contributions

KY: supervision and resources. IF-L: formal analysis and methodology. JR and NG: data curation. All authors contributed to the conceptualization of the study and writing of the original draft, and have approved the final manuscript.

## Conflict of Interest

The authors declare that the research was conducted in the absence of any commercial or financial relationships that could be construed as a potential conflict of interest.

## Footnotes

^1^Sources: Open Data Austria. Retrieved on July 15, 2020 from: www.data.gv.at/covid-19; Assessment Capacities Project (ACAPS). Retrieved on July 15, 2020, from www.acaps.org

^2^Since the second-order Akaike's Information Criterion (AIC_C_) was slightly lower for variable selection step 7 (AIC_C_ = 101.86) compared to step 6 (AIC_C_ = 102.15), the factor age was removed by the automated backward variable selection procedure in SPSS in the final step (data available upon request). However, the correct classification rate in step 7 was 66.7%, which is lower than in step 6 (70.5%). Given the negligible difference in AIC_c_ scores, the model with the highest classification rate was favored in this analysis.
